# Early Post–cesarean Section Gangrenous Ileocecal Volvulus in a Primigravida: A Rare Postpartum Complication Case Report

**DOI:** 10.1002/ccr3.72726

**Published:** 2026-05-18

**Authors:** Anteneh Girma Mengistu, Nathanael Elias Temesgen, Selamawit Kassahun Aweke, Natnael Mathewos Assale, Bemnet Ashenafi Kebede

**Affiliations:** ^1^ Department of Surgery Addis Ababa University College of Health Sciences, Tikur Anbessa Hospital Addis Ababa Ethiopia; ^2^ Addis Ababa University College of Health Sciences, Tikur Anbessa Hospital Addis Ababa Ethiopia

**Keywords:** case report, cesarean section complication, ileocecal region, intestinal obstruction, postpartum period, volvulus

## Abstract

Cecal volvulus is a rare cause of intestinal obstruction, especially in the postpartum period. It occurs when the cecum, terminal ileum, and ascending right colon twist around an axis, which leads to obstruction, ischemia, and possible gangrene. We report a rare case of early postcesarean gangrenous ileocecal volvulus in a 21‐year‐old mother, just two days after an emergency cesarean section. The patient showed right‐sided abdominal swelling, pain, vomiting, and absence of bowel movements. Imaging showed dilated bowel loops with air–fluid levels, indicating a small‐bowel obstruction. An exploratory laparotomy confirmed a 540° clockwise ileocecal volvulus with gangrenous changes, requiring resection and double‐barrel ileocolostomy. The patient had a smooth recovery after the surgery. This case highlights the need to be alert for cecal volvulus in postpartum women who have sudden abdominal distention following a cesarean delivery, as early identification and quick surgical intervention are essential to avoid serious complications.

## Key Clinical Message

Cecal volvulus should be considered in early postpartum women who have unexplained abdominal swelling or pain suggesting obstruction. Early recognition and quick surgical treatment are crucial to prevent gangrene, sepsis, and death. Delayed diagnosis can make outcomes much worse.

## 1 Introduction

Cecal volvulus is a rare clinical condition characterized by axial twisting of the cecum, terminal ileum, and ascending right colon around their mesenteric pedicle. The main pathophysiologic mechanism is cecal hypermobility associated with precipitating factors such as colonic tumor, abdominal mass, or pregnancy. Cecal volvulus has been reported during pregnancy; importantly, a recent systematic review found that roughly 25% of volvulus cases present in the early postpartum period, underscoring the need to consider this diagnosis in postpartum patients with compatible symptoms [1].

Cecal volvulus and pregnancy were reported in several publications with an incidence ranging from 1/2500 to 1/3500. Maternal mortality differs by sites (12.5% for cecal versus 5.5% for sigmoid volvulus), and although maternal mortality was not affected by timing of delivery relative to surgery, fetal mortality was substantially higher when the interval was < 24 h, 52.9% vs. 10.4%. Both maternal and fetal mortality have decreased over time [1].

Increased uterine volume may explain cecal volvulus during pregnancy. In the postpartum period, it may be explained by rapid uterine‐size variation. Cecal volvulus diagnosis is challenging. Its symptoms can be mistaken for postoperative ileus or for the even more common postpartum Ogilvie syndrome (acute colonic pseudo‐obstruction) after cesarean section delivery, which occurs more frequently in postpartum patients than in young nonpregnant postoperative patients [2]. Early recognition is crucial, as gangrenous transformation can lead to peritonitis and sepsis within 24–48 h, necessitating urgent surgical intervention.

Surgical options in the treatment of cecal volvulus include colonic detorsion, colonic detorsion and either cecopexy or cecostomy, and right hemicolectomy [3].

This case report presents a rare case of postpartum gangrenous ileocecal volvulus in a 21‐year‐old primigravida just two days after an emergency cesarean section, highlighting the importance of a high index of suspicion in postcesarean section abdominal symptoms as well as the need for timely management to prevent complications.

## 2 Case History and Examination

A 21‐year‐old woman was transferred from the maternity ward to our surgical emergency unit with a 2‐day history of right‐sided abdominal distension that began approximately 12 h after cesarean section. Associated with this, she also experienced abdominal pain, distention, nausea, vomiting, and obstipation, along with a fever of the same duration. Two days ago, she underwent an emergency cesarean section at GA of 41 + 6 weeks due to Grade 2 meconium‐stained amniotic fluid and fetal tachycardia during her first pregnancy, resulting in the delivery of a 2.4‐kg live female neonate with an APGAR score of 7 and 8 at 1 and 5 min, respectively.

After her initial presentation, she was kept in the maternity ward with the diagnosis of postoperative ileus, but the symptom did not improve and she was later transferred to our unit.

Upon her arrival, a physical examination revealed that the patient was tachycardic (heart rate of 136 bpm), febrile (temperature of 38.3°C), and tachypenic (respiratory rate of 30 breaths per minute), with an oxygen saturation of 95% on room air and normal blood pressure of 127/82 mmHg. During the general examination, she appeared acutely ill but alert, with a grossly distended abdomen prominent on the right side. There was generalized abdominal tenderness, marked on the right side, and absent bowel sounds in the right lower quadrant. A digital rectal examination revealed an empty rectum. There was a clean transverse surgical incision in the suprapubic area; otherwise, she had no history of chronic constipation, hernias, or history of appendectomy.

## 3 Differential Diagnosis, Investigations and Treatment

Postcesarean bowel injury/perforation (ileum or cecum) and acute postoperative ileus progressing to early SBO were entertained differentials considered besides ileocecal/cecal volvulus.

### 3.1 Blood Tests Revealed

- White blood cell (WBC) count: 14.3 × 10^3^/μL (normal range: 3.98–10.04 × 10^3^/μL)—elevated (leukocytosis).
- Neutrophil count: 89.7%—elevated (neutrophilia).
- Hemoglobin level: 12 g/dL (normal range: 11.2–15.7 g/dL)—within normal limits.
- Mean cell volume (MCV): 87.4 fL (normal range: 79.4–94.8 fL)—within normal limits.
- Platelet count: 272,000/μL (normal range: 182–369/μL)—within normal limits.

### 3.2 Biochemical Investigations

- Sodium: 137 mmol/L (normal range: 136–145 mmol/L)—within normal limits.
- Potassium: 3.64 mmol/L (normal range: 3.5–5.5 mmol/L)—within normal limits.
- Chloride: 106 mmol/L (normal range: 98–107 mmol/L)—within normal limits.
- Urea: 30 mg/dL (normal range: 7–18 mg/dL)—elevated.
- Creatinine: 0.4 mg/dL (normal range: 0.6–1.3 mg/dL)—low.
- Lactate dehydrogenase (LDH): 298 IU/L (normal range: 91–180 IU/L)—elevated.
- Coagulation profile: Normal.

**FIGURE 1:**
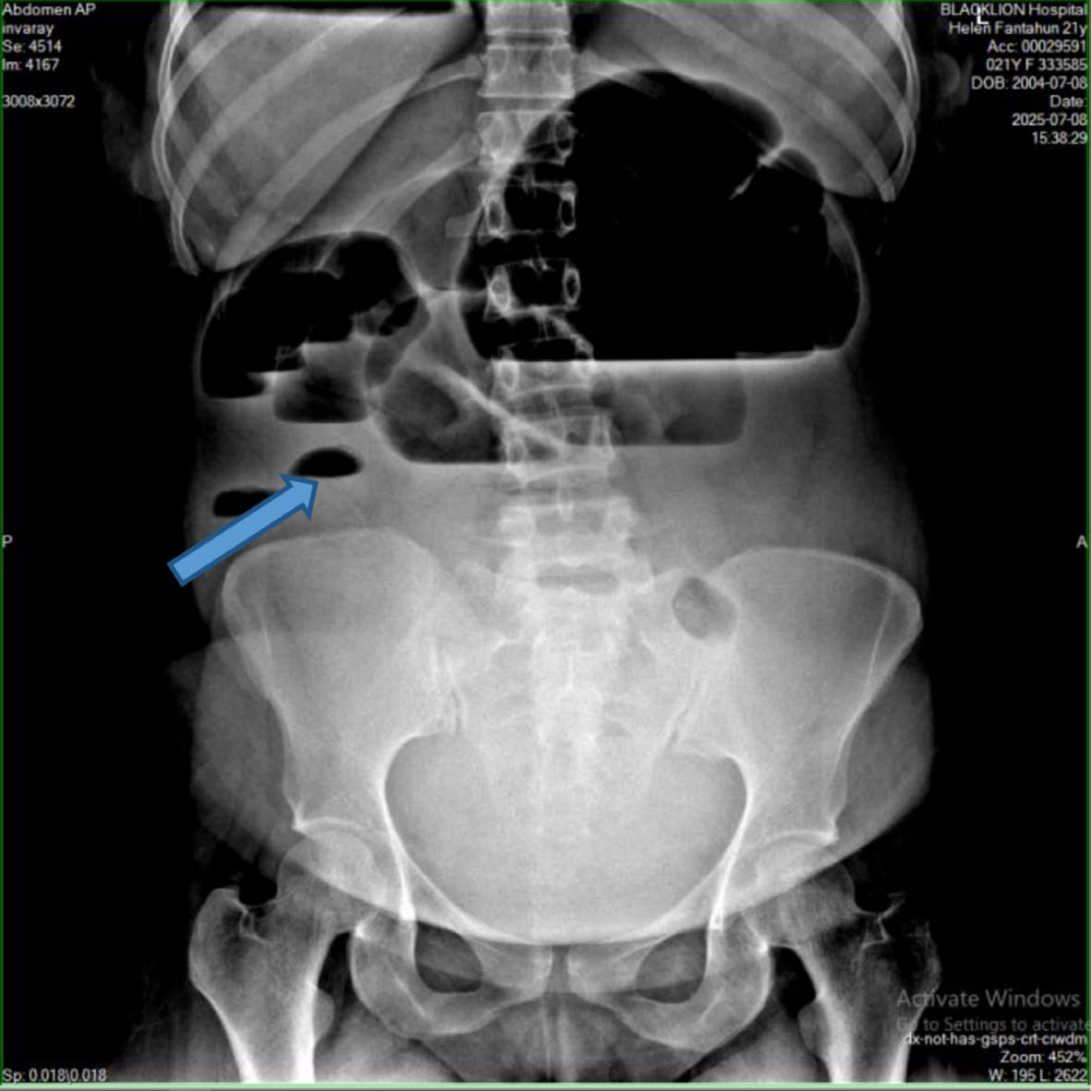
PA erect abdominal X‐ray shows multiple dilated small‐bowel loops with prominent air–fluid levels (blue arrow) and minimal colonic gas, consistent with small‐bowel obstruction.

PA erect abdominal X‐ray was ordered and showed (Figure 1) multiple dilated bowel loops exceeding normal diameter, with air–fluid levels at different heights. The dilated bowel loops are primarily in the small intestine, indicated by their central location and the presence of valvulae conniventes (folds crossing the full width of the bowel), which suggest small‐bowel obstruction. There was absent gas in the rectum.

After the patient was resuscitated and started on empirical metronidazole 500 mg and ceftriaxone 1 g intravenously in the surgical emergency unit, written informed consent was obtained and she was taken to the main operating room for exploratory laparotomy under general anesthesia for the diagnosis of small‐bowel obstruction.

Through a midline vertical incision, the peritoneum was entered. Intraoperatively, approximately 300 mL of thin pus was found in the general peritoneum. The cecum was hugely distended with patchy necrosis. The cecum was hypermobile, and there was an ileocecal volvulus of 540 degrees in a clockwise direction. The appendix appeared normal with a pink serosal surface, no evidence of hyperemia, edema, or perforation. The proximal bowel was dilated and edematous. The uterus was approximately the size of a 16‐week pregnancy, and the cesarean section site was intact.

**FIGURE 2:**
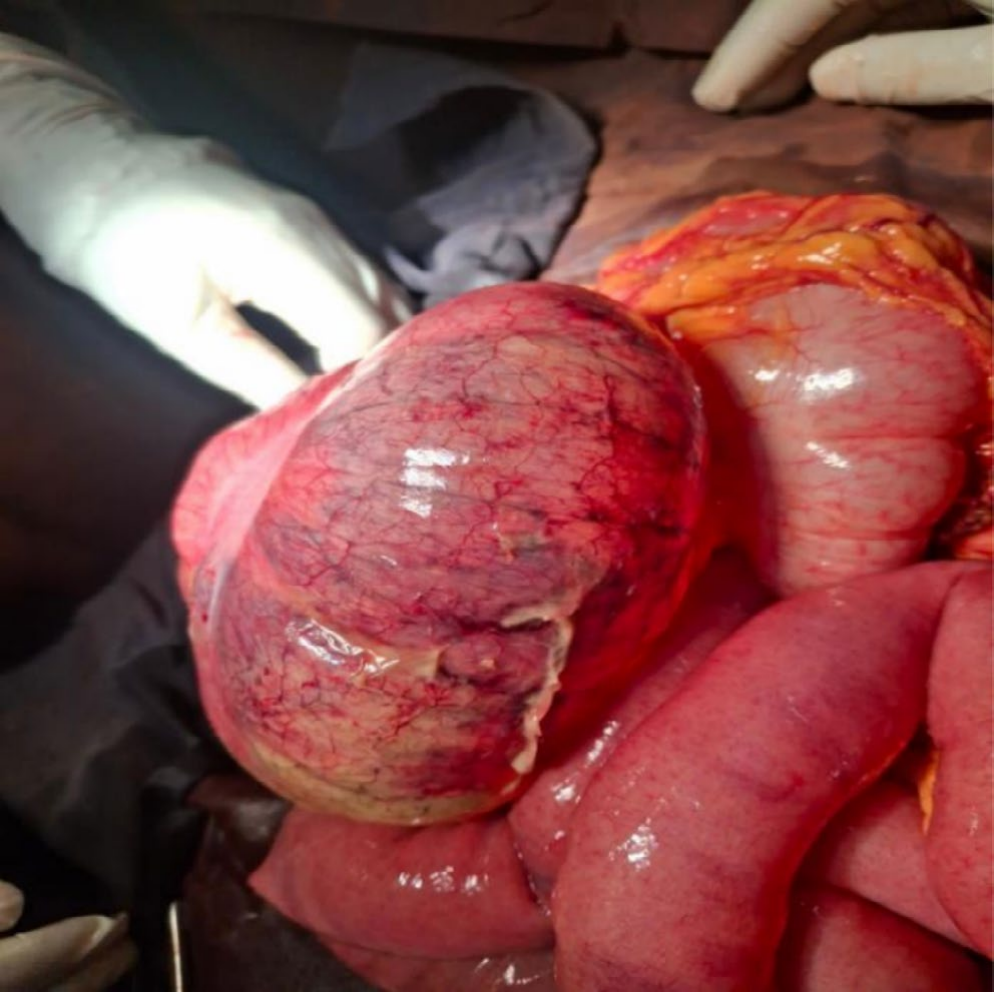
Hugely distended hyper‐mobile edematous cecum with patchy necrosis and 540° clockwise ileocecal volvulus.

**FIGURE 3:**
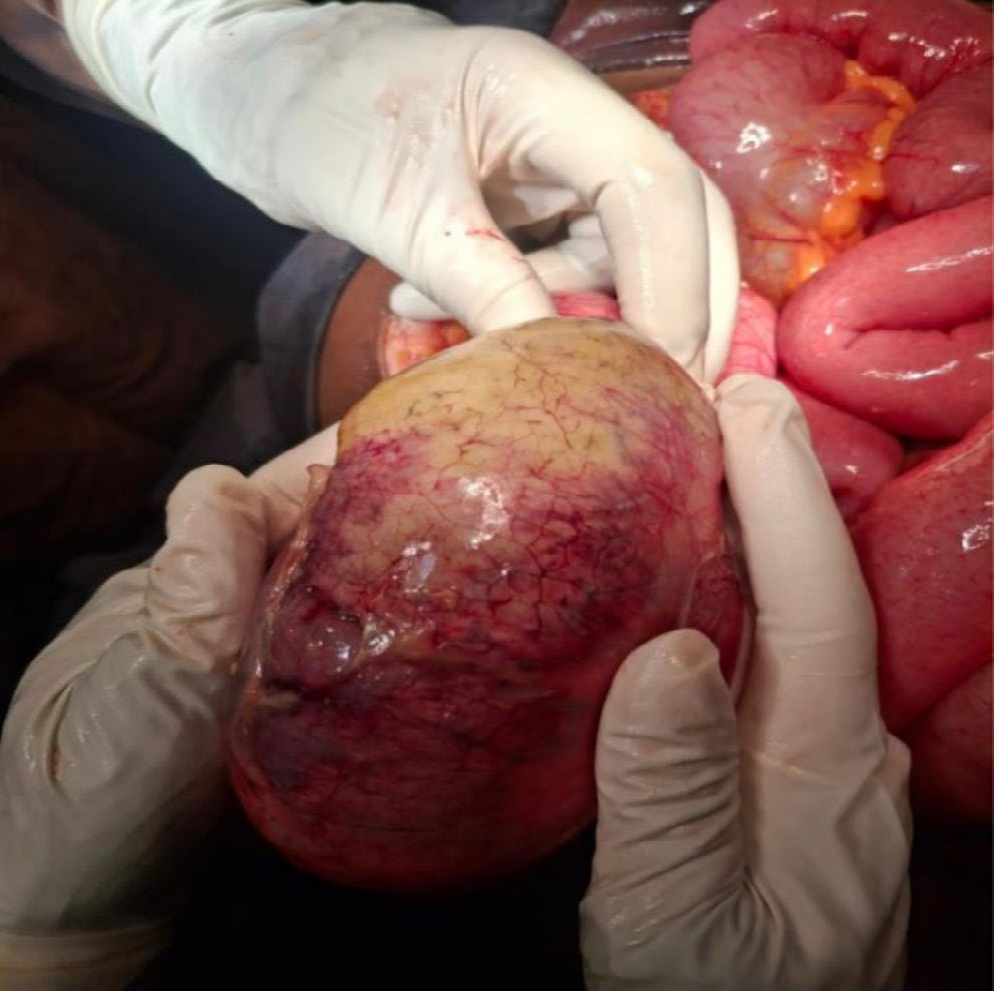
Different view of hugely distended hyper‐mobile edematous cecum with patchy necrosis and 540° clockwise ileocecal volvulus.

With these intraoperative findings (Figures 2 and 3), gangrenous ileocecal volvulus was definitively diagnosed.

Thin pus was suctioned out, and 10 cm of the terminal ileum and cecum was resected. The terminal ileum and ascending colon were brought out as a double‐barrel ileocolostomy. The abdomen was lavaged with warm normal saline. The fascia was closed in a continuous manner, and the skin was closed and dressed, and the patient was extubated on the table and transferred to the postanesthesia care unit with stable vital signs. On her first day postoperatively, metronidazole 500 mg IV TID and ceftriaxone 1 g IV BID were continued.

However, two weeks after her postoperative period, she developed right lower quadrant abdominal pain and a fever of 36.1°C. Abdominal ultrasound was performed at the time of her presentation, and a right lower mesenteric, loculated, and septated echocomplex fluid collection measuring about 10 × 6 × 5 cm was appreciated. The complete blood count (CBC) revealed a white blood cell (WBC) count of 11.1 × 10^3^/μL, with a neutrophil percentage of 64%. Ultrasound‐guided abscess drainage was performed successfully. She was admitted, and the same antibiotics were continued for another two weeks empirically. (An abscess culture was not performed because the patient could not afford the investigation.)

## 4 Outcome and Follow‐Up

She has shown a complete clinical improvement after an overall stay of one month, which was prolonged due to intermittent fever and complete antibiotic therapy. She was discharged with a scheduled follow‐up appointment after three months. Three months after her discharge, ileostomy reversal was performed as planned, and she started oral intake on her 24th postoperative hour. She was discharged after 7 days with a smooth recovery. A chronological summary of key events, including symptoms, investigations, treatments, and outcomes, is shown in Table 1.

**TABLE 1:** Historical and current information of care organized as a timeline.

Time point/day	Event/intervention	Clinical findings/notes
Day 0	Emergency cesarean section (C/S)	Indication: Grade 2 meconium‐stained amniotic fluid, fetal tachycardia; delivered 2.4 kg female neonate, APGAR 7 and 8 at 1 and 5 min. C/S site clean, no immediate complications.
Day 2 (Post‐C/S)	Onset of symptoms/ED presentation	Right‐sided abdominal distention, pain, nausea, vomiting, no passage of stool or gas, fever 38.3°C. Vitals: HR 136 bpm, RR 30, BP 127/82, SpO_2_ 95%. Abdominal exam: generalized abdominal tenderness, marked on the right side with absent bowel sounds
	Laboratory and Imaging	Labs: leukocytosis 14.3 × 10^3^/μL, neutrophilia 89.7%: dilated small‐bowel loops, air–fluid levels, absent rectal gas. Ultrasound: dilated bowel loops, minimal fluid in Morrison's pouch.
	Exploratory laparotomy	Midline incision; 300 mL of pus in peritoneum; cecum hypermobile, distended, patchy necrosis; ileocecal volvulus 540° clockwise. Intervention: resection of 10‐cm terminal ileum + cecum, double‐barrel ileocolostomy, abdominal lavage.
Postoperative Day 1	Postop care	Started IV antibiotics: metronidazole 500 mg TID, ceftriaxone 1 g BID. Patient stable, extubated, transferred to PACU.
Postoperative Day 14 (2 weeks)		Right lower quadrant pain, mild fever (36.1°C). Ultrasound: 10 × 6 × 5 cm loculated mesenteric fluid collection (abscess).
Complication		Ultrasound‐guided drainage; continued antibiotics for the next 2 weeks. Clinical improvement noted.
		Admitted for next 2 weeks and managed as an inpatient.
1 Month postop	Discharge	Patient discharged in stable condition; follow‐up appointment in 3 months scheduled.
3 Months postop	Ileostomy reversal	Successful reversal of ileocolostomy; discharged after 7 days with smooth recovery.

## 5 Discussion

Cecal volvulus (CV) is the axial rotation of the ascending colon, cecum, and terminal ileum, which was first described by Rokitansky in 1837 [4]. It is a rare condition accounting for 1%–2% of all large‐bowel obstructions [5]. The cecum is the second‐most common site of colonic volvulus after the sigmoid colon [6]. The suggested mechanism of cecal volvulus associates cecal hypermobility to a precipitating factor like pregnancy [7], uterine leiomyoma [8], or colonic tumor.

The clinical presentation depends on the duration of the complaints and the presence of complications. Symptoms of abdominal pain, associated with vomiting and abdominal distension, are reported as the commonest presentations [9]; all the mentioned symptoms were documented in our patient. Preoperative diagnosis of CV poses a significant challenge because of its rarity and nonspecific symptoms; it is mostly diagnosed intraoperatively [10].

Laboratory investigations are neither specific nor sensitive for the diagnosis of CV but may suggest the degree of obstruction and the presence of complications [11]. Our patient had an elevated leukocytosis level with a predominant neutrophil count, which can be explained by the presence of a strangulated cecum.

Radiologic imaging may be abnormal and detect CV in 45%–56% of the cases. Plain abdominal X‐ray is highly sensitive for the diagnosis of CV with the characteristic “coffee bean” sign deformity, its apex pointing to the left upper quadrant [12]. Other findings commonly seen are cecal dilatation (98%–100%), single air–fluid level (72%–88%), small‐bowel dilatation (42%–55%), and the absence of gas in the distal colon (82%). All the mentioned radiologic signs were present in our patient. CT is more sensitive and specific for diagnosing CV and detecting complications [10]. CT showed a markedly dilated, malpositioned cecum with a whirl sign and abrupt transition to collapsed distal colon, and focal wall hypoenhancement and pericecal fat stranding suggesting closed‐loop obstruction with early ischemia.

The cecal volvulus management is predominantly surgical. Conservative management consists of detorsion and cecal fixation; however, the preferred treatment is right hemicolectomy with primary anastomosis or ileostomy [6, 8]. The morbidity is about 20%, and the mortality is very variable depending on the series, varying between 9% and 60%. The worst outcomes of CV are explained by management delay [13]. In our case, the management was surgical, and an ileocolostomy was performed.

## 6 Conclusion

Cecal volvulus is a rare postpartum complication after a cesarean section. Delayed management leads to a worse prognosis. Early clinical suspicion is needed for early diagnosis and management to decrease further complications.

## Author Contributions

**Nathanael Elias Temesgen:** conceptualization, investigation, writing – original draft. **Anteneh Girma Mengistu:** conceptualization, investigation, writing – original draft. **Selamawit Kassahun Aweke:** validation, writing – original draft, writing – review and editing. **Natnael Mathewos Assale:** validation, writing – original draft, writing – review and editing. **Bemnet Ashenafi Kebede:** conceptualization, investigation, writing – original draft.

## Funding

The authors have nothing to report.

## Ethics Statement

Ethics approval is not required for a single anonymous case at our institution.

## Consent

Written informed consent was obtained from the patient for publication of this case report and any accompanying images. A picture of the consent is added on the end of this manuscript and a clear pdf is available to share on request.

## Conflicts of Interest

The authors declare no conflicts of interest.

## Data Availability

All the data generated or analyzed during this study are included in this published article.
